# Incidence and risk factors for peripheral intravenous catheter failure in Japanese neonatal intensive care units and growing care units: a multicenter observational study

**DOI:** 10.20407/fmj.2025-027

**Published:** 2026-02-28

**Authors:** Shun Yamamoto, Ryoko Murayama

**Affiliations:** 1 Graduate School of Health Sciences, Fujita Health University, Toyoake, Aichi, Japan; 2 Department of Nursing, Faculty of Health Science, Nagoya Aoi University, Nagoya, Aichi, Japan; 3 Faculty of Nursing, School of Health Sciences, Fujita Health University, Toyoake, Aichi, Japan; 4 Research Center for Implementation Nursing Science Initiative, Fujita Health University, Toyoake, Aichi, Japan

**Keywords:** NICU, Catheter failure, Peripheral intravenous catheter, Multicenter observational study, Japan

## Abstract

**Objectives:**

Peripheral intravenous catheters (PIVCs) are used in neonatal intensive care units (NICUs) and growing care units (GCUs). However, neonates/infants in these units frequently experience catheter failure (CF) because of complications, such as extravasation and phlebitis. Nonetheless, data on PIVCs from Japan remain limited. Therefore, this study aimed to investigate the use of infusion catheters and the incidence, characteristics, and risk factors of PIVC-related catheter failure in Japanese NICUs and GCUs.

**Methods:**

This study was conducted between August 2024 and February 2025 using cross-sectional and prospective surveys. Data from 600 hospitalized neonates/infants and 259 PIVC cases were analyzed. CF was defined as unplanned catheter removal before the completion of therapy owing to loss of function or an inability to administer fluids.

**Results:**

Overall, 48.0% of neonates/infants had infusion catheters. The incidence proportion of CF was 47.1%, with an incidence density of 173.1/1,000 catheter-days. Extravasation was the most frequently identified cause of CF (95.9%), and swelling and leaking were primary symptoms. Multivariate Cox regression showed that gestational age (hazard ratio=0.96, p=.03) and infusion flow volume at the time of PIVC removal (hazard ratio=1.28/1 mL/h, p<.01) were significant risk factors. Long PIVCs had significantly longer dwell times than PIVCs (log-rank p=.01).

**Conclusions:**

CF is highly prevalent in Japanese infants in NICUs/GCUs, with a rate similar to that reported internationally. Long PIVCs may help extend the catheter dwell times. These findings highlight the need for standardized prevention strategies and improved catheter management protocols.

## Introduction

Neonates and infants hospitalized in neonatal intensive care units (NICUs) and growing care units (GCUs) (hereafter referred to as infants) often experience difficulties in establishing enteral nutrition owing to physiological immaturity or underlying medical conditions. Consequently, infants require intravenous fluid therapy for hydration, nutritional support, medication administration, and blood product transfusions. Available vascular access options include peripherally inserted central catheters (PICCs), central venous catheters, umbilical venous catheters, and peripheral intravenous catheters (PIVCs). Although PICCs, central venous catheters, and umbilical venous catheters are instrumental for long-term parenteral nutrition, including total parenteral nutrition, their placement is highly invasive and associated with risks of infection and thrombosis.^1–3^ In contrast, PIVCs are less invasive than these other catheters and thus considered essential, making them the most commonly used form of vascular access in neonatal intensive care. However, placement and maintenance of PIVCs are technically challenging, particularly for infants.^4,5^

The vasculature and integument of infants are immature relative to those of adults, with more fragile vessels and underdeveloped skin integrity. In infants, systemic instability and heightened susceptibility to infection exacerbate the clinical effect of vascular and cutaneous immaturity. The 24-gauge (24G) catheter, with an outer diameter of approximately 0.55 mm, is commonly used for PIVC insertion in infants because it has a diameter similar to that of dorsal hand veins (0.7–1.8 mm), which are a frequent cannulation site. The mechanical irritation caused by the catheter increases as the blood vessel decreases in size.^6^ Although PIVC-mediated intravenous fluid therapy is indispensable for life-sustaining care in infants, it is associated with a high risk of catheter failure (CF) and complications, such as occlusion, extravasation, and phlebitis, which may cause early removal before treatment is completed.^7–11^

Previous studies have reported that approximately 40%–65% of infants with PIVC experience CF, primarily because of extravasation or occlusion, often accompanied by clinical signs, such as infiltration or phlebitis.^7–11^ However, detailed studies regarding the incidence, causes, and clinical manifestations of PIVC-related CF in infants in Japan are lacking. Although CF incidence rates in Japan may be similar to those reported internationally, differences in medical materials, securement methods, and healthcare delivery systems limit the direct applicability of international findings to the Japanese context.

Therefore, the present study aimed to clarify the current use of infusion catheters and determine the incidence and clinical characteristics of PIVC-related CF among infants hospitalized in the NICU and GCU in Japan. This study will contribute to the development of prevention strategies for CF by identifying the principal risk factors for CF in Japan and delineating targeted interventions to address these factors.

## Methods

### Design and setting

This study used anonymized data collected from August 2024 to February 2025. A survey was requested from 150 core accredited institutions and 16 designated accredited institutions under the subspecialty certification system of the Japan Society of Perinatal and Neonatal Medicine. These facilities were selected because of their well-established infrastructure, robust physician and nurse staffing, and high inpatient volumes. The data were collected using three purpose-specific questionnaires. Questionnaires 1 and 3 were from cross-sectional studies, whereas Questionnaire 2 was from a prospective cohort study.

Questionnaire 1 was completed by a representative of each participating facility and captured information about all infants who were hospitalized in the NICU or GCU on a day designated by the facility representative. The primary outcome was the proportion of hospitalized infants who received intravenous fluid therapy. Secondary outcomes included hospitalized ward type (NICU/GCU), gestational age, postnatal age in days, body weight, presence of an infusion catheter, catheter types, indwelling needle types, and gauge of the indwelling needle.

Questionnaire 2 was a prospective data collection tool completed by nurses in the NICU or GCU wards of the participating facility over 1 month, starting from a designated date. Nurses recorded data on infants for whom a new PIVC was inserted and subsequently removed during the data collection period. The primary outcome was the incidence of CF in infants using a PIVC. Secondary outcomes comprised the date and time of PIVC insertion, gestational age, postnatal age, body weight, purpose of infusion, use of a long peripheral intravenous catheter (LPIVC), type and gauge of indwelling needle, profession of the inserter, number of insertion attempts, insertion site, use of insertion aids, date and time of PIVC removal, profession of the remover, reason for removal, symptoms and signs at removal, presence and site of medical device-related pressure injuries, flow volume, content of infusion at removal, and whether re-insertion of a PIVC was performed. The reason for PIVC removal and any symptoms or signs that were present at the time of the removal were left to the discretion of the nurses present during the PIVC removal procedure. If a PIVC was placed multiple times within a month, the times of placement and removal were recorded to ensure that it was identified for the same individual. In Questionnaires 1 and 2, the exclusion criterion was a request for study exclusion by the infants’ families before data collection.

Questionnaire 3 was completed by a representative of each facility and provided information regarding the presence and content of clinical protocols for infusion management in the wards.

### Definition of terms

In this study, in accordance with Murayama et al.,^12^ catheter failure was defined as the unplanned removal of a PIVC before completion of the intended therapy because of loss of function or inability to administer fluid.

### Procedure

The designated representative (e.g., director of nursing and head nurse of the NICU/GCU) at each target institution was contacted by phone to inform them of the impending dispatch of documents requesting their participation in the study.

Thereafter, a comprehensive package of materials was mailed and included the study protocol and a formal letter requesting research collaboration detailing the study’s objectives and clinical significance, methodology, anticipated benefits and potential risks, assurance of no disadvantage for nonparticipation, procedures for handling infants’ data, and conflict of interest disclosures. Additionally, the package included a reply form for confirming institutional willingness to collaborate, the three types of questionnaires, an opt-out information document for infants’ families, and an institutional approval form for study implementation. This material was then mailed back after the representatives responded. Facilities that did not return the survey forms were reminded of this lack of return.

### Data analysis

Statistical analyses were conducted using JASP software (version 0.19.3.0; Amsterdam, The Netherlands) and Python (version 3.12.11) and R (version 4.5.1, fisher.test function) on Google Colaboratory. Descriptive statistics, including the median and range, were used to summarize the data. The assumption of a normal distribution for continuous variables was assessed using the Kolmogorov–Smirnov test. When a non-normal distribution was indicated for these variables, medians and ranges were reported, and non-parametric tests were used for intergroup comparisons. The Mann–Whitney U and Kruskal–Wallis tests were used for comparisons between two and three or more independent groups, respectively. When significant differences were observed, post-hoc multiple comparisons were performed using the Dunn–Bonferroni method. Comparisons between nominal variables were performed using the χ^2^ test or Fisher’s exact test. The χ^2^ test was used when the expected frequency of each cell was ≥5, and Fisher’s exact test was used when the expected frequency of each cell was <5. The χ^2^ test was performed, and if significant differences were found, a residual analysis was performed for intergroup comparisons. A Cox proportional hazards model was used to identify the risk factors associated with CF. Variables that showed an association with CF at p<.10 in univariate analyses (Mann–Whitney U or χ^2^ test) were considered for inclusion as covariates in the Cox proportional hazards model. Hazard ratios (HRs) and their corresponding 95% confidence intervals (CIs) were calculated using the Cox proportional hazards model. Two-sided p<.05 was considered statistically significant. The goodness-of-fit of the Cox proportional hazards model was evaluated using the likelihood ratio test. The Cox proportional hazards model indicated a trend towards significance (p<.10) for the association between the use of an LPIVC and CF. Therefore, Kaplan–Meier curves were generated to examine the differences in PIVC dwell times. The log-rank test was used to compare survival curves between relevant groups.

### Ethical considerations

This study was conducted in strict adherence to the ethical principles outlined in the Declaration of Helsinki and complied with the Ethical Guidelines for Life Science and Medical Research Involving Human Subjects stipulated in Japan. The study protocol was approved by the Ethics Review Board of Fujita Health University (approval no. HM24-031). When required by institutional policy, local ethical approval was obtained following appropriate procedures. Taking into consideration that the participants were neonates or infants, an information disclosure document pertaining to this research was prominently displayed in a location readily accessible to the participants’ families, thereby ensuring an opportunity for them to opt out of participation.

## Results

### Participants

**Figure 1:**
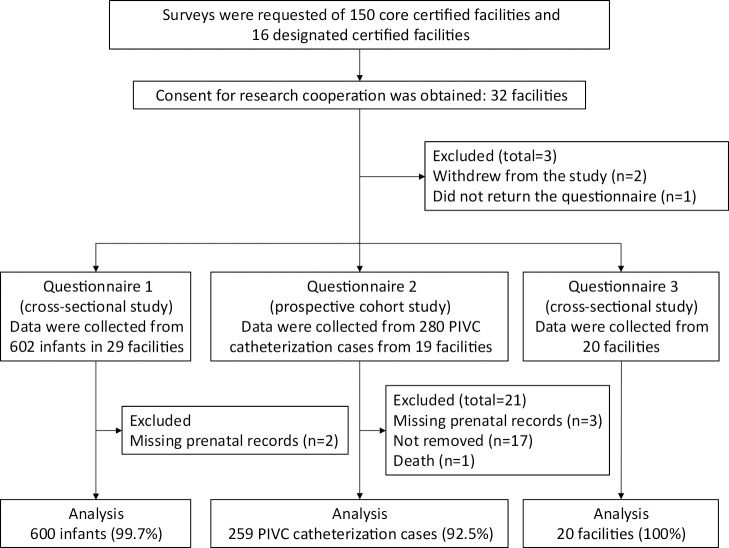
Flow of each questionnaire in the study Number of facilities and infants included in the analysis, with exclusions and final sample sizes for each questionnaire.

The overall flow, including the number of facilities and infants included, exclusions, and the final numbers analyzed per questionnaire, of the enrolled infants is shown in Figure 1.

### Characteristics of the infants and catheter use for infusion therapy

Data were collected from 602 infants in 29 facilities on a specific day. Two infants were excluded because of a lack of prenatal obstetric records, which made determining their gestational age impossible. Consequently, 600 (99.7%) infants were included in the analysis.

The median weight of the infants was 2,450 g (range, 334–12,810 g), median gestational age was 36 weeks (range, 22–43 weeks), and median age was 15 days (range, 0–1,303 days). Of these, 288 (48.0%) had an infusion catheter, of whom 249 (86.5%) were hospitalized in the NICU and 39 (13.5%) in the GCU. In infants aged 0–2 days, 126 of 149 (84.0%) had an infusion catheter. The proportion of infants with an infusion catheter placed was 87.6% (85/97) on postnatal day 0 and 86.7% (117/135) in infants aged 0–1 day. Table 1 shows the infants’ characteristics. The most commonly used catheter type was a PICC, followed by a PIVC and an 8-cm PICC. Regarding the 8-cm PICC, whether it was used as an LPIVC or a PICC is unclear. In the NICU, 52 (20.9%) infants had 2 or more types of infusion catheters concurrently, whereas in the GCU, all infants had only 1 type. The proportion of infants with multiple catheter types was significantly higher in the NICU than in the GCU (p<.01).

Infants with an infusion catheter were categorized into three groups according to body weight (≤1,499, 1,500–2,499, and ≥2,500 g, Table 2). The proportion of infants with a PIVC significantly increased with increasing body weight (p<.01). In the ≤1,499 g group, PICC use was significantly higher than that in the other body weight groups (p<.01), and PICCs were used more frequently than PIVCs. There was no significant difference in the number of concurrent infusion catheter types between the body weight groups (p=.20).

### Incidence of CF and symptoms/signs at infusion catheter removal

Data were collected from 280 cases of PIVC catheterization during the study period from 19 facilities. Twenty-one cases were excluded because of the following reasons: (1) the lack of prenatal obstetric visits and unconfirmed gestational age, (2) the PIVC was not removed during the study period, or (3) death. A total of 259 (92.5%) cases were included in the analysis.

CF occurred in 122 of the 259 cases, resulting in an incidence rate of 47.1%. The CF incidence density was 173.1 events per 1,000 catheter-days. The main reasons and symptoms/signs for removal before completing infusion therapy in the 122 cases with CF are shown in Table 3.

The most common reason for catheter removal was extravasation followed by occlusion. Although catheter replacement and dislodgement/accidental removal were reported in one (0.8%) case, these were excluded from this study because they did not correspond to CF. The most frequent symptoms and signs of CF observed were swelling, followed by leaking and redness. Other findings included resistance during flushing, induration, infusion pump alarms, skin problems, arm discoloration, bleeding from the insertion site, and increased C-reactive protein concentrations.

### Occurrence of CF and associated factors

The factors associated with the occurrence of CF in infants with indwelling PIVCs are shown in Table 4. The indwelling needles were 24G for all infants. During the 1-month survey period, 40 infants had a PIVC placed more than twice, with a maximum number of five times. The maximum number of needle punctures required for PIVC placement was six. Although a vein visualization device was used in approximately 70% of PIVC insertions, ultrasonography was not used before catheter placement in any case. Five facilities used an LPIVC.

In the univariate analysis, infants in the CF group had a significantly shorter gestational age (p=.01) and significantly shorter catheter dwell time than the non-CF group (p<.01). In the multivariate Cox proportional hazards model, the HR for CF per 1-week increase in gestational age was 0.96 (95% CI: 0.92–1.00, p=.03). This finding corresponded to an approximately 1.04-fold increase in the risk of CF for each 1-week decrease in gestational age.

**Figure 2:**
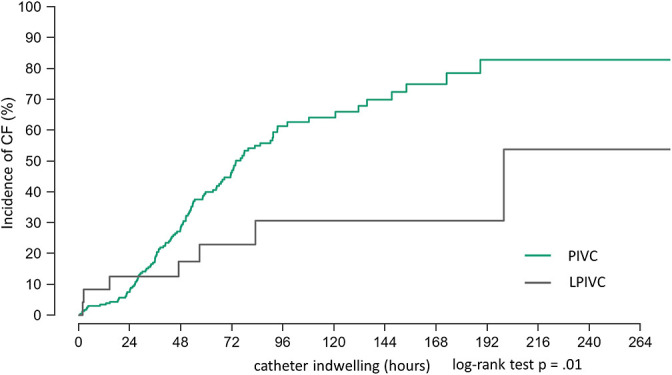
Kaplan-Meier curves for PIVC and LPIVC dwell times Comparison of standard peripheral intravenous catheters (PIVCs; green line) with long peripheral intravenous catheters (LPIVCs; grey line). The x-axis represents the catheter indwelling time in hours, and the y-axis represents the cumulative incidence of CF (%). A comparison of CF-free survival distributions using the log-rank test showed a significant difference between the two catheter types (p=.01).

Although the use of an LPIVC was not significant in the univariate analysis (p=.07), its tendency towards significance warranted inclusion in the multivariate analysis. The multivariate analysis showed a non-significant trend towards a reduced risk of CF (HR=0.47, 95% CI: 0.22–1.04, p=.06). Although the multivariate analysis did not show significant results, we further compared the cumulative incidence trends of CF between PIVCs and LPIVCs over time using the Kaplan–Meier method. Kaplan–Meier curves comparing the cumulative CF incidence over time between the PIVC and LPIVC groups are shown in Figure 2. The PIVC group showed an earlier and higher incidence of CF, with a widening difference over time. At 72 h post-insertion, the cumulative incidence of CF was approximately 45% and 25% in the PIVC and LPIVC groups, respectively. After 96 h, these values were approximately 60% and 30%, respectively. There was a significant difference in the time-to-event distributions between the groups (log-rank test, p=.01).

The median flow volume of infusion at the time of catheter removal was significantly higher in the CF group than in the non-CF group in the univariate analysis (p<.01). In the multivariate analysis, each 1 mL/h increase in infusion flow volume was associated with a 1.28-fold higher risk of CF (HR=1.28, 95% CI: 1.20–1.37, p<.01).

Other variables, such as postnatal age, body weight, purpose of infusion, profession of the inserter, number of insertion attempts, insertion site, use of insertion aids, contents of infusion at removal, and presence of medical device-related pressure ulcers, were not significantly associated with CF.

### PIVC management protocols and fixation materials at each facility

Data were collected from 20 facilities. All facilities have established institutional protocols for managing PIVCs. The main content documented in the manuals included the indications for PIVC use (n=8), insertion techniques (n=10), securement methods (n=20), observation procedures (n=16), removal criteria (n=2), and the removal method (n=9). Three facilities reported using guidelines for PIVC placement, with one specifically referencing the Centers for Disease Control and Prevention guidelines. Seventeen facilities reported not having a standard procedure for PIVC removal. Transparent film dressings (19 facilities), splints (19), elastic tape (8), nonelastic tape (8), nonwoven tape (13), and protective interface materials (e.g., gauze or base layer) between the catheter and the skin (17) were used to secure the PIVC.

## Discussion

To the best of our knowledge, this study is the first large-scale multicenter investigation of the current use of infusion catheters in Japan, the incidence of PIVC-related CF, and factors associated with PIVC-related CF in the NICU and GCU settings. At the time of data collection, 288 (48.0%) infants had infusion catheters in place. Among the infants aged 0–2 days, 126 (84.0%) had infusion catheters inserted, which suggested that nearly all infants in the NICU or GCU required some form of infusion catheter during their hospitalization. Additionally, this study showed that the proportion of infants who weighed ≤1,499 g and had a PICC was significantly higher than that in those who had a heavier weight. Similarly, the proportion of infants who weighed ≥2,500 g and had a PIVC was significantly higher than that in those with a lower body weight. In very low birth weight infants, PICC placement is preferable because it accommodates the need for long-term parenteral nutrition and drug administration while reducing the frequency of venous punctures, thereby preserving peripheral veins.^13,14^ In contrast, in infants weighing ≥2,500 g, a PIVC is the preferred option because it is less invasive and provides short-term fluid therapy.

The incidence of CF in this study was 47.1%, which fell within the range of 40%–65% reported in previous international studies.^7–11^ The CF incidence density was 173.1 events per 1,000 catheter days, which is higher than that (113.3–136.0 events per 1,000 catheter days) reported in other studies that included pediatric and adult patients.^15–17^ This high rate may be attributed to the anatomical characteristics of infants, particularly the small caliber and fragility of their peripheral veins.

Extravasation was the cause of >95% of the CF cases, and swelling and leaking were the most frequently reported symptoms. While the proportion of symptoms was similar to that reported in previous studies,^7–11,18^ the proportion of CF caused by extravasation was higher in our study. This discrepancy between studies may be attributed to the fact that, while these previous studies showed that extravasation and phlebitis were the main causes of CF, this study reported no cases of phlebitis. In Questionnaire 3, few facilities set criteria for when to remove PIVCs. Therefore, the criteria for determining phlebitis differed within facilities, and catheters may have been removed early because of symptoms of phlebitis, such as swelling and leaking.

The gestational age at the time of PIVC placement and infusion flow volume at the time of PIVC removal were identified as risk factors for CF. No significant association was found between body weight and CF in this study. However, in a previous study, a lower body weight at PIVC insertion was associated with a risk of CF,^7^ which is consistent with our results, because a shorter gestational age was associated with a lower body weight. In infants, anatomical factors, such as fine and fragile vessels and immature skin, combined with mechanical irritation from body movement and difficulties in catheter securement, contribute synergistically to an increased risk of CF. Our finding that a higher infusion flow volume was associated with an increased risk of CF suggests that mechanical irritation of the vascular endothelium and pressure load from syringe pumps or infusion pumps contribute to the development of CF. Therefore, in infants requiring high-volume infusions, the use of a PICC or LPIVC should be considered to reduce the risk of CF.

A comparison of the Kaplan–Meier curves showed that CF occurred earlier and more frequently in infants with a PIVC than in those with an LPIVC, with this difference increasing over time, which is consistent with previous studies.^19,20^ Therefore, in infants in whom infusion therapy exceeds 48 h, replacing a PIVC with an LPIVC may reduce the risk of CF and contribute to more stable treatment continuity and developmental outcomes. Furthermore, careful observation is required for approximately 48 h after PIVC placement because the risk of developing CF is high.

This study showed variations in managing PIVCs and the materials used for fixation among facilities, suggesting a lack of standardized PIVC management protocols. Previous studies have indicated that securement methods and prevention of infection are particularly crucial in PIVC management.^4,21^ Therefore, the development and implementation of standardized protocols and evidence-based guidelines are essential for reducing the incidence of CF in the future. None of the facilities in this study reported ultrasonography during PIVC insertion. In the pediatric field, the success rate of PIVC placement has been increased by using ultrasonography,^22^ and blood vessels on the dorsum of the hand and foot of neonates can also be imaged using this method.^6^ Based on these findings, even in the neonatal field, selecting thicker, straighter blood vessels under ultrasonography before placing a PIVC may be an effective strategy to reduce mechanical stimulation and prevent CF of PIVCs in infants.

This study has some limitations. First, unmeasured confounding variables, such as disease and severity, detailed methods of catheter fixation, and detailed contents of infusion, may have affected the outcomes. Second, because there are no established, standardized criteria for determining CF, the decision was left to the discretion of the professionals at each facility. Consequently, this lack of criteria may have introduced a reporting bias (e.g., variation in CF classification criteria). Finally, this study was conducted in facilities with well-established infrastructure and staff capable of providing advanced medical care. These centers typically treat infants with higher acuity who may be more prone to adverse events. Therefore, caution should be exercised when generalizing these findings to all NICUs and GCUs in Japan. Based on these limitations, future studies should aim to standardize materials and fixation methods for PIVC placement. Future studies should also conduct comparative analyses across institutions differing in size and with different severities of the infants’ condition to better understand the risk of CF and prevention strategies.

## Conclusion

In this large-scale, multicenter study in Japan, we found a high incidence of PIVC-related catheter failure in infants hospitalized in NICUs and GCUs. A shorter gestational age and higher infusion flow volumes at the time of PIVC removal were significant risk factors for CF. Our findings indicate that the use of LPIVCs could reduce CF, particularly for infusions expected to last >48 h. Our findings suggest the need for standardized PIVC management protocols to improve the quality of intravenous fluid therapy and outcomes for this vulnerable population.

## Figures and Tables

**Table 1:** Infants’ characteristics and status of indwelling catheters in NICUs and GCUs

	Total n=288	NICU n=249	GCU n=39	NICU vs GCU p value
Body weight (g)	2,441 (334–9,590)	2,379 (334–9,590)	2,860 (1,030–3,894)	<.01
Gestational age (weeks)	36 (22–42)	36 (22–42)	38 (24–41)	<.02
Postnatal age (days)	4 (0–1,303)	4 (0–1,303)	6 (0–445)	.16
Catheter type				
PIVC	137 (47.6)	120 (48.2)	17 (43.6)	.59
PICC	169 (58.7)	150 (60.2)	19 (48.7)	.17
8-cm PICC	9 (3.1)	8 (3.2)	1 (2.6)	1.00
CVC	9 (3.1)	7 (2.8)	2 (5.1)	.35
UVC	5 (1.7)	5 (2.0)	0 (0.0)	1.00
UAC	5 (1.7)	5 (2.0)	0 (0.0)	1.00
PAC	22 (7.6)	22 (8.8)	0 (0.0)	.05
Number of catheter types				<.01
One	236 (81.9)	197 (79.1)	39 (100.0)	
Two	37 (12.8)	37 (14.9)	0 (0.0)	
Three	14 (4.9)	14 (5.6)	0 (0.0)	
Four	1 (0.3)	1 (0.4)	0 (0.0)	
Gauge of the indwelling needle				
24G	183 (63.5)	158 (63.5)	25 (64.1)	
26G	0 (0.0)	0 (0.0)	0 (0.0)	
Others	124 (43.1)	110 (44.2)	14 (35.9)	

Data are presented as the median (minimum–maximum) or as n (%). The p values for body weight, gestational age, and postnatal age were analyzed using the Mann–Whitney U test. The p values for catheter type (8-cm PICC, CVC, UVC, UAC, and PAC) and the number of catheters were analyzed using Fisher’s exact test, and other categorical variables were analyzed using the χ^2^ test. PIVC: peripheral intravenous catheter; PICC: peripherally inserted central catheter; CVC: central venous catheter; UVC: umbilical venous catheter; UAC: umbilical arterial catheter; PAC: peripheral arterial catheter; G: gauge. Whether the 8-cm PICC was used as a long PIVC or a PICC is unclear.

**Table 2:** Infants’ characteristics in relation to body weight

	≤1,499 g n=67	1,500–2,499 g n=83	≥2,500 g n=138	p value
Gestational age (weeks)	29 (22–38)	36 (23–39)	39 (26–42)	<.01 ^a, b, c^
Postnatal age (days)	10 (0–166)	1 (0–445)	3 (0–1,303)	<.01 ^a^
Catheter type				
PIVC	15 (22.4)	42 (50.6)	80 (58.0)	<.01 ^i, iii^
PICC	55 (82.1)	48 (57.8)	66 (47.8)	<.01 ^i, iii^
8-cm PICC	4 (6.0)	1 (1.2)	4 (2.9)	.33
CVC	0 (0.0)	2 (2.4)	7 (5.1)	.14
UVC	4 (6.0)	1 (1.2)	0 (0.0)	<.01
UAC	4 (6.0)	1 (1.2)	0 (0.0)	<.01
PAC	6 (9.0)	6 (7.2)	10 (7.2)	.92
Number of catheter types				.16
One	49 (73.1)	69 (83.1)	118 (85.5)	
Two	15 (22.4)	10 (12.0)	12 (8.7)	
Three	3 (4.5)	4 (4.8)	7 (5.1)	
Four	0 (0.0)	0 (0.0)	1 (0.7)	

Data are presented as the median (minimum–maximum) or as n (%). The p values for gestational age and postnatal age were analyzed using the Kruskal–Wallis test, and post hoc multiple comparisons were conducted using Dunn’s test with the Bonferroni method. The p values for catheter type (PIVC, PICC) were compared using the χ^2^ test, and residual analysis was performed for between-group comparisons when appropriate. The p values for other categorical variables were analyzed using Fisher’s exact test. ^a^ p<.01 for ≤1,499 g vs 1,500–2,499 g, ^b^ p<.01 for ≤1,499 g vs ≥2,500 g, ^c^ p<.01 for 1,500–2,499 g vs ≥2,500 g. ^i^ p<.01 for ≤1,499 g vs other groups, ^ii^ p<.01 for 1,500–2,499 g vs other groups, ^iii^ p<.01 for ≥2,500 g vs other groups. PIVC: peripheral intravenous catheter; PICC: peripherally inserted central catheter; CVC: central venous catheter; UVC: umbilical venous catheter; UAC: umbilical arterial catheter; PAC: peripheral arterial catheter.

**Table 3:** Reasons for CF and symptoms and signs

	n=122
Reasons for CF	
Extravasation	116 (95.1)
Occlusion	6 (4.9)
Symptoms and signs for CF	
Swelling	63 (51.6)
Leaking	55 (45.1)
Redness	22 (18.0)
Induration	3 (2.5)
Resistance	2 (1.6)
Pump alarm	2 (1.6)
Skin problems	2 (1.6)
Phlebitis	0 (0.0)
Other	3 (2.5)

Data are presented as n (%). CF: catheter failure.

**Table 4:** Cox proportional hazards analysis of the presence and cause of CF

	Non-CF n=137	CF n=122	Univariate p value	Multivariate		
				HR	95% CI	p value
Gestational age (weeks)	38 (23.0–41.9)	37 (24.6–41.7)	.01	0.96	(0.92–1.00)	.03
Postnatal age (days)	1 (0–146)	1 (0–187)	.98			
Body weight (g)	2,678 (751–5,536)	2,489 (631–8,365)	.21			
≤1,499 g	8 (5.8)	12 (9.8)				
1,500–2,499 g	49 (35.8)	51 (41.8)				
≥2,500 g	80 (58.4)	59 (48.4)				
Catheter dwell time (h)	63.5 (7.5–450.0)	47.5 (0.8–199.9)	<.01			
Purpose of infusion						
PPN	112 (81.8)	97 (79.5)	.65			
Antibiotics	41 (29.9)	40 (32.8)	.62			
Inspection	17 (12.4)	10 (8.2)	.27			
Blood transfusion	3 (2.2)	5 (4.1)	.48			
Other	8 (5.8)	6 (4.9)	.74			
Use of a long PIVC	17 (12.4)	7 (5.7)	.07	0.47	(0.22–1.04)	.06
Profession of the inserter			.10			
Physician	137 (100)	119 (97.5)				
Nurse	0 (0.0)	3 (2.5)				
Number of insertion attempts			.46			
First	73 (53.3)	72 (59.0)				
Second	37 (27.0)	25 (20.5)				
≥Third	27 (19.7)	25 (20.5)				
Insertion site			.68			
Dorsum of the hand	93 (67.9)	81 (66.4)				
Cephalic vein	19 (13.9)	21 (17.2)				
Basilic vein	6 (4.4)	7 (5.7)				
Median antebrachial vein	3 (2.2)	0 (0.0)				
Dorsum of foot	7 (5.1)	8 (6.6)				
Small saphenous vein	3 (2.2)	2 (1.6)				
Great saphenous vein	6 (4.4)	3 (2.5)				
Use of insertion aids						
No use	40 (29.2)	39 (32.0)	.63			
Vein visualization device	97 (70.8)	82 (67.2)	.53			
Ultrasonography	0 (0.0)	0 (0.0)				
Contents of infusion at catheter removal			.62			
PPN	102 (74.5)	89 (73.0)				
Saline solution	25 (18.2)	20 (16.4)				
Other	10 (7.3)	13 (10.7)				
Flow volume of infusion at catheter removal (ml/h)	1 (0.0–15.0)	2.9 (0.0–12.0)	<.01	1.28	(1.20–1.37)	<.01
MDRPU	5 (3.6)	3 (2.5)	.73			

Data are presented as the median (minimum–maximum) or as n (%). The p values for gestational age, postnatal age, body weight, catheter dwell times, and flow of infusion at catheter removal were analyzed using the Mann–Whitney U test. The p values for the purpose of infusion (blood transfusion), profession of the inserter, insertion site, and MDRPU were analyzed using Fisher’s exact test. The p values for other categorical variables were analyzed using the χ^2^ test. Variables with p<.10 by the Mann–Whitney U test or χ^2^ test were entered into the Cox proportional hazards analysis. The fit of the model was p<.001 by the likelihood test. CF: catheter failure; PIVC: peripheral intravenous catheter; PPN: peripheral parenteral nutrition; MDRPU: medical device-related pressure ulcer.
